# Investigation of factors potentially influencing calcitonin levels in the screening and follow-up for medullary thyroid carcinoma: a cautionary note

**DOI:** 10.1186/1472-6890-13-27

**Published:** 2013-11-04

**Authors:** Christoph Guesgen, Arnulf Willms, Axel Zwad, Stephan Waldeck, Helmut Wieler, Robert Schwab

**Affiliations:** 1 Department of General, Visceral and Thoracic Surgery, German Armed Forces Central Hospital, Ruebenacher Strasse 170, Koblenz 56072, Germany; 2 Department of Radiology, German Armed Forces Central Hospital, Ruebenacher Strasse 170, Koblenz 56072, Germany; 3 Department of Nuclear Medicine, German Armed Forces Central Hospital, Ruebenacher Strasse 170, Koblenz 56072, Germany

**Keywords:** Calcitonin, Medullary thyroid carcinoma, Calcitonin screening, Goitre, Thyroid, Proton pump inhibitor, Hashimoto’s thyroiditis

## Abstract

**Background:**

The malignant transformation of thyroid C cells is associated with an increase in human calcitonin (hCT), which can thus be helpful in the early diagnosis of medullary thyroid carcinoma (MTC). For this reason, hCT levels should be determined in all patients with nodular goitre. Hashimoto’s thyroiditis, nodular goitre and proton pump inhibitor (PPI) therapy are factors reported to influence basal serum hCT concentrations. The diagnostic role of mildly to moderately increased hCT levels is thus a matter of debate. In this study, we attempt to clarify the role of the aforementioned factors.

**Methods:**

From 2008 to 2009, we collected data from 493 patients who were divided into five groups. We assessed whether there were significant differences in hCT levels between patients with Hashimoto’s thyroiditis, patients with nodular goitre, patients with PPI therapy, and healthy control subjects. In addition, we investigated whether a delayed analysis of blood samples has an effect on serum hCT concentrations.

**Results:**

Immunoradiometric assays (Calcitonin IRMA magnum, MEDIPAN) revealed that the time of analysis did not play a role when low levels were measured. Delayed analysis, however, carried the risk of false low results when serum hCT concentrations were elevated. Men had significantly higher serum hCT levels than women. The serum hCT concentrations of patients with Hashimoto’s thyroiditis and nodular goitre were not significantly different from those of control subjects. Likewise, PPI therapy did not lead to a significant increase in serum hCT concentrations regardless of the presence or absence of nodular goitre.

**Conclusions:**

Increases in serum hCT levels are not necessarily attributable to Hashimoto’s thyroiditis, nodular goitre or the regular use of PPIs and always require further diagnostic attention.

## Background

Medullary thyroid carcinoma (MTC) is a malignant tumor of the thyroid gland that represents 1,4% - 10% of all thyroid carcinomas [1].

It develops from the crest-derived parafollicular C-cells and exists in 2 forms: sporadic and familial. Metastases spread via the lymphatic system. The sporadic form represents 75% of the MTCs. In the recent literature, the mean prevalence of sporadic MTC was found to be 0.18 – 0.4% of all patients with thyroid nodules [2]–[6]. Approximately 25% of all MTCs occur as the result of the autosomal dominant syndromes MEN and familial MTC [7]. Both syndromes are caused by distinct germline mutations in the RET proto-oncogene encoding a transmembrane receptor with cytoplasmatic tyrosine kinase activity.

The malignant transformation of thyroid C cells is associated with an increased production of human calcitonin as a result of a dysfunction of the regulatory system. For this reason, the measurement of calcitonin levels is a useful tool for the early detection, diagnosis and follow-up of MTCs. Since the early detection of MTCs is associated with excellent prospects for cure and MTCs – like all highly differentiated tumours – mostly tend to grow slowly, early diagnosis and treatment play an important role despite the low prevalence of MTC. [8].

Human calcitonin (hCT) is a peptide hormone that consists of 32 amino acids and is produced in humans by the parafollicular cells (C cells) of the thyroid. It is part of a regulatory system and helps control serum concentrations of calcium. Bones, the kidneys and the gastrointestinal tract are the main targets of the biolocigal effects of calcitonin. Evidence of interactions between C cells and thyroid cells suggest that there is a functional relationship between these types of cells although there is still a lack of precise data [9].

Serum contains only very low levels of hCT. There are no ethnic differences in basal serum hCT concentrations but men are reported to have higher concentrations than women [2,10]–[12].

Patients with clinically apparent MTC usually have serum hCT levels that are 10 to 100 times higher than normal [13,14]. Markedly elevated basal serum hCT levels or pentagastrin-stimulated serum hCT levels higher than 100 pg/ml are thus indicative of MTC. At postoperative follow-up, such levels may suggest a recurrence or untreated metastases [11,13,14]. Normal serum hCT concentrations range from 0 to 10 pg/ml for women and from 0 to 15 pg/ml for men [15]. Pentagastrin and calcium are the usual provocative agents used worldwide. Both tests are performed in patients with nodular thyroid disease and mildly elevated basal serum calcitonin concentrations. At the moment pentagastrin is no more available in several countries, therefore the intravenous calcium stimulation test is used more often.

In the literature, Hashimoto’s thyroiditis, nodular goitre and the use of proton pump inhibitors (PPIs) have been reported to influence basal serum hCT concentrations [3,15]–[20]. If, for example, patients are intolerable to pentagastrin and cannot undergo a pentagastrin stimulation test for an evaluation of increased serum hCT levels, omeprazole can be used instead to induce an increase in serum hCT concentrations [16]. However the omeprazole test has not been validated yet. Further studies are required to investigate the influence of a regular use of PPIs [19].

Patients with moderately elevated hCT levels, i.e. levels that are no more than 10 times higher than normal, are difficult to evaluate, especially in the absence of clinical evidence and the presence of potential influencing factors that have been described in the literature. For this reason, the diagnostic value of mildly or moderately increased hCT levels is currently being discussed in the literature [20]. A concrete evaluation of these levels is required.

The objective of the present study was to investigate factors possibly influencing serum calcitonin concentrations. We assessed whether gender, Hashimoto’s thyroiditis, nodular goitre or the regular use of PPIs influences serum hCT levels and whether a delayed analysis of blood samples has an effect on serum hCT measurements. We conducted this study in an attempt to shed more light on the role of mildly or moderately increased serum calcitonin levels.

## Methods

### Study design and patients

This prospective single-centre study included 493 consecutive patients (406 men and 87 women) with Hashimoto’s thyroiditis or nodular goitre with or without PPI therapy who attended the thyroid and surgical clinics of the German Armed Forces Central Hospital in Koblenz over a period of two years (from 2008 to 2009).

Diagnosis of Hashimoto’s thyroiditis was based on laboratory tests including measurements of free triiodothyronine (fT3), free thyroxine (fT4), thyroid-stimulating hormone (TSH), microsomal antibodies (MAB), and thyroglobulin antibodies (TAB), as well as on the clinical picture and ultrasonography. A typical clinical presentation is a painless diffuse enlargement of the thyroid gland accompanied by hypothyoidism. A typical ultrasound scan shows a generalized hypoechogenicity usually of the entire thyroid gland [21]–[23]. Marked heterogeneity of the internal structure is seen in 70% of the patients [24]. A proportion of patients shows hypoechoic pseudonodular and multifocal lesions representing areas of high inflammatory activity, i.e. lymphocytic infiltration [25].

The control group consisted of volunteers with a normal thyroid who did not take PPIs and were attending the surgical outpatient clinic. Regular use of PPIs was defined as the daily intake of a minimum dose of 10 mg of omeprazole, 20 mg of pantoprazole or 10 mg of esomeprazole over a period of at least two weeks.

Patients with a history of radioiodine treatment or thyroid surgery, patients with malignant or severe non-malignant systemic diseases (≥ ASA 3 according to the American Society of Anaesthesiologists classification system), and patients who had undergone surgery involving another organ system during the previous six months were excluded from the study. Further exclusion criteria were known hypercalcitoninaemia, renal insufficiency, bacterial infection, known alcohol abuse, and pseudohypoparathyreodism.

Patient assessments included a medical history, a physical examination, and thyroid ultrasonography. In addition, laboratory tests were performed to measure the levels of fT3 (pg/ml), fT4 (pmol/l), TSH (μIE/ml), CEA (ng/ml) and calcitonin (pg/ml).

On the basis of their data, patients were assigned to one of five groups for analysis.

Group 1 : patients with Hashimoto’s thyroiditis who did not take PPIs (n = 122).

Group 2 : patients with nodular goitre and PPI therapy (n = 73).

Group 3 : patients with nodular goitre who were not treated with PPIs (n = 118).

Group 4 : patients with a normal thyroid and with PPI therapy (n = 59).

Group 5 : patients with a normal thyroid who did not take PPIs (n = 121, control group).

The study was conducted in compliance with the Helsinki Declaration and was approved by our local ethics committee (Ethics Committee, Medical Council Rhineland-Palatinate, Germany, IRB00004206). All patients or their legal representatives gave their written informed consent.

### Laboratory tests

Serum levels of thyroid parameters were determined no later than 60 minutes after sampling in the Laboratory of Nuclear Medicine of the German Armed Forces Central Hospital in Koblenz. If this was impossible, serum samples were frozen at −20°C. Serum hCT concentrations were determined using an immunoradiometric assay (Calcitonin IRMA magnum, MEDIPAN; normal levels range from 0 pg/ml to 15 pg/ml for men and from 0 pg/ml to 10 pg/ml for women) [26].

This assay is highly specific and highly sensitive and has a functional sensitivity of 1.5 pg/ml [26]. In accordance with the manufacturer’s instructions, venous blood samples were processed no later than two hours after sampling or frozen at −20°C since otherwise there was a risk of false low results. For an assessment of the influence of delayed serum analysis on calcitonin levels, a series of 20 randomly selected serum samples was analysed immediately after sampling and after storage for two and four hours at room temperature.

### Statistical analysis

We compared the means and medians for the five groups in order to detect any significant differences and to assess the influence of Hashimoto’s thyroiditis, nodular goitre or the regular use of PPIs on serum hCT concentrations. All data were entered into an Excel® spreadsheet and analysed using SPSS® Version 15.0. The level of significance was set at p < 0.05.

First, we performed an analysis of variance (ANOVA) for a single factor, i.e. mean serum hCT concentrations. If significant differences between the means were found, we used a post-hoc Bonferroni test for multiple comparisons of dependent means.

For a gender-specific analysis of serum hCT concentrations and a comparison of the different patient groups, we determined not only means but also medians in order to minimise the effects of outliers. Statistical analysis was performed using a Kruskal and Wallis H test. If the H test showed significant between-group differences, a Mann–Whitney U test was used to identify groups that were significantly different from each other.

## Results

### General characteristics

The mean age of the patients was 48 years (range: 17–87 years). 482 patients (97,9%) were euthyroid, 11 (2,1%) were hypothroid and no patient was hyperthyroid.

The mean serum hCT concentration was 6.385 pg/ml (± 5.7845 pg/ml) for all subjects. A comparison of serum hCT levels for different age groups did not reveal remarkable differences. 191 patients had a nodular goiter. Ultrasound detected multiple nodules in 156 patients (82%) and a solitary nodule in 35 patients (18%). The mean diameter of the dominant nodule in the patient group with multiple nodules was 19 ± 4 mm (range: 7–34 mm). The mean diameter of the nodules in the patient group with solitary nodules was 22 ± 3 mm (range: 10–42 mm).

### Influence of time of blood sample analysis

Statistically, there was a mean difference of 1.40 pg/ml between the levels measured immediately and after storage for two hours at room temperature and 1.42 pg/ml between the levels measured immediately and after storage for four hours. The mean difference between the levels measured after two hours and after four hours was as low as 0.023 pg/ml. With p-values of 0.749 and 0.726 respectively, the mean differences of 1.40 pg/ml and 1.42 pg/ml were statistically not significant. The mean difference of the measured hCT concentrations was below the functional assay sensitivity of approximately 1.5 pg/ml specified by the manufacturer. It should be noted that several calcitonin levels that were mildly elevated (>7 pg/ml, n = 7) during the initial measurement decreased considerably. This difference, however, was not significant.

### Influence of gender

The male subjects had significantly higher mean concentrations (p = 0.0001) and median concentrations of serum hCT (p = 0.0001) than the female subjects. In male subjects (n = 406) we measured mean serum hCT concentrations of 6.876 pg/ml (± 5.85 pg/ml) and median serum hCT concentrations of 6.055 pg/ml (± 5.85 pg/ml). In female subjects the mean serum hCT concentrations were 4.094 pg/ml (± 4.91 pg/ml) and the median serum hCT concentrations 2.720 pg/ml (± 4.91 pg/ml).

### Analysis of patient groups

Table 1 provide an overview of the mean and median serum hCT concentrations for the five patient groups. There were significant differences between both the mean (p = 0.012) and median concentrations (p = 0.01) of the patient groups. The group of patients with Hashimoto’s thyroiditis showed the lowest mean concentration, i.e. 5.368 pg/ml (± 5 pg/ml), and the lowest median concentration, i.e. 4.12 pg/ml (± 5 pg/ml). By contrast, the group of patients with a normal thyroid and PPI therapy had the highest mean concentration, i.e. 8.672 pg/ml (± 9.447 pg/ml), and the highest median concentration, i.e. 6.1 pg/ml (± 9.447 pg/ml).

**Table 1:** Mean and median serum hCT concentrations for the different patient groups

**Patient group**	**Number**	**Median**	**Mean**	**Standard deviation**
Group 1: Hashimoto’s thyroiditis	122	4.120	5.368	5.0051
Group 2: nodular goitre and PPI therapy	73	6.060	6.341	3.9486
Group 3: nodular goitre without PPI therapy	118	5.740	6.212	4.0923
Group 4: normal thyroid and PPI therapy	59	6.100	8.672	9.4468
Group 5: normal thyroid without PPI therapy	121	5.200	6.491	6.2806
Total	493	5.350	6.385	5.7845

### Influence of Hashimoto’s thyroiditis, nodular goitre and PPI therapy

**Figure 1:**
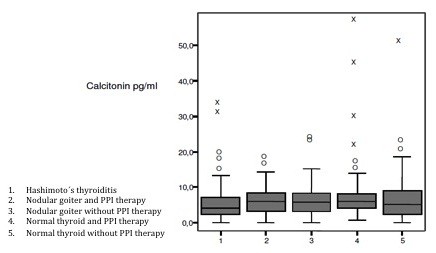
**Box plots displaying the 25th and 75th percentiles, the medians, the whiskers extending from the 2.5th to the 97.5th percentile for the different groups of patients (O** = **increase, X** = **outlier).**

An analysis of the mean concentrations for the different patient groups revealed no significant differences between Groups 1 to 4 and Group 5 (control subjects with a normal thyroid and no PPI therapy) (Figure 1, Table 1). The only significant difference (p = 0.004) was found between the mean concentrations for patients with Hashimoto’s thyroiditis (Group 1) and patients with a healthy thyroid and PPI therapy (Group 4). The mean concentration for Group 1 was 5.368 pg/ml (± 5 pg/ml) and was thus significantly lower than that for Group 4, which was 8.672 pg/ml (± 9.447 pg/ml). All other differences between the mean concentrations for the various groups were not significant.

An analysis of the median concentrations showed no significant differences between Groups 1 to 4 and Group 5 (subjects with a normal thyroid and no PPI therapy) (Figure 1, Table 1).

Significant differences, however, were found between the group of patients with Hashimoto’s thyroiditis and all other groups with the exception of the control group (normal thyroid without PPI therapy). Patients with Hashimoto’s thyroiditis showed a significantly lower median serum hCT concentration than Group 2 patients (nodular goitre with PPI therapy) (p = 0.014), Group 3 patients (nodular goitre without PPI therapy) (p = 0.012), and Group 4 patients (normal thyroid and PPI therapy) (p = 0.001).

There were no further significant differences between the medians for the patient groups. The maximum difference between median concentrations, however, was 1.98 pg/ml and was thus only slightly above the functional assay sensitivity of approximately 1.5 pg/ml as specified by the manufacturer.

The box plots in Figure 1 show a high number of outliers for Group 4 (normal thyroid and PPI therapy) that resulted in a just significant difference between the means and medians of Groups 1 and 4 (Figure 1) and an increased standard deviation for Group 4.

## Discussion

Since even very small MTCs tend to metastasise [27] and prognosis depends on early diagnosis and treatment [6,28,29], which consists of thyroidectomy and lymphadenectomy [30], early detection and management play an important role despite the low prevalence of MTC. The prospects for cure are excellent after surgery if treatment is instituted at an early stage, i.e. before MTC has metastasised. A 10-year survival rate of 97.7% has been reported [31]. Because of the high sensitivity and specificity of serum hCT measurement by immunoassay technology using two monoclonal antibodies routine hCT measurement has been suggested for assesment of patients with thyroid nodular disease by major authorities in Europe [2,20,32,33] and the United States [34,35]. Yet it is not rountinely performed in most cases. In a survey of Bennebaek et al. the serum hCT measurement was only used in 43% of all cases with nodular thyroid disease [36]. However the routine use of serum hCT screening in patients with nodular thyroid disease is still under debate [5]. There are also several authors, who do not recommend the routine use because of the hight prevelance of thyroid nodules and the rarity of MTC. Routine hCT measurement has no general acceptance in the US [37]. Therefore the expert panel of the ATA (American Thyroid Association) could not recommend either for or against the routine measurement of serum calcitonin in 2009 (Recommendation rating: I) [38]. Despite the previous AACE-AME guidelines did not endorse the routine measurement of hCT the revised 2010 guidelines favors, but does not recommend, routine hCT testing: “measurement of basal serum calcitonin level may be useful in the initial evaluation of thyroid nodules” [39].

The European Thyroid Association (ETA) and the German Society of Endocrinology (DGE) recommend the routine hCT screnning in patients with nodular thyroid disease [14,15,30].

Patients with clinically apparent MTC usually have serum hCT levels that are 10 to 100 times higher than normal. Markedly elevated hCT levels are thus indicative of MTC. At postoperative follow-up, such levels may suggest a recurrence or untreated metastases [28]. Patients with mildly or moderately elevated hCT levels (not exceeding 100 pg/ml) are difficult to evaluate, especially since the literature reports a number of factors that can influence serum calcitonin concentrations [3,8,15,18]. Hypercalcitoninaemia can occur with a broad spectrum of conditions. An elevation of calcitonin levels can be found not only in patients with MTC but also in patients with C-cell hyperplasia (CCH), which is difficult to differentiate and may precede MTC. In addition, up to 22% of patients with renal failure present with markedly elevated serum hCT concentrations [27]. In the literature, moderate elevations in serum hCT levels (not exceeding 100 pg/ml) were also reported in patients receiving proton pump inhibitor (PPI) therapy [16,17,40] as well as in patients with Hashimoto’s thyroiditis [18,19].

Likewise, hypercalciuria [41], paraneoplastic syndromes [15], and chronic alcoholism [42] were found to induce an increase in hCT levels. By contrast, there is no evidence to support earlier research suggesting that elevated hCT levels are caused by medicines containing calcitonin or salmon calcitonin [15,26] or elevated procalcitonin levels associated with bacterial infections [26], which were found by modern highly specific and sensitive assays without cross-reactivities to be largely insensitive to the aforementioned influences.

The interpretation of mildly or moderately elevated serum hCT concentrations always requires that the risk of surgery for a benign condition be weighed against the risk of missing an MTC. For this reason, the diagnostic role of serum hCT concentrations is a matter of debate [20].

When the test kit (Calcitonin IRMA magnum) was used in this study to compare immediate and delayed analyses of hCT levels, there were no significant differences in the results when low levels within normal limits were measured. A delayed analysis of blood samples with hCT levels that were primarily elevated but still within normal limits led to a few lower values after two hours at room temperature. This suggests that false low results can indeed be produced in the case of elevated hCT concentrations and a delayed analysis. Accordingly, valid results can only be obtained if the processing steps and times specified by the manufacturer of the assay are strictly observed [26].

The present results confirm the finding that men have significantly higher serum hCT concentrations than women. Saller et al. and Vierhapper et al., too, reported gender-specific differences and measured higher basal serum hCT concentrations in men [2,12]. According to the manufacturer of the immunoradiometric assay used in this study, normal calcitonin levels in the serum of healthy persons range from 0 pg/ml to 15 pg/ml in men and from 0 pg/ml to 10 pg/ml in women [26]. Elevated serum hCT concentrations in women must receive particular attention since a medullary thyroid carcinoma is the underlying cause in approximately 80% of the cases [2]. By contrast, moderately elevated serum hCT concentrations in men are the result of C-cell hyperplasia in up to 80% of the cases [2].

The maximum difference between mean levels was 3.304 pg/ml (maximum standard deviation: 9.4468). This is mainly attributable to the considerable number of outliers observed for Group 4 (healthy thyroid and PPI therapy). When a statistical analysis of the medians was performed in order to minimize the influence of outliers, the maximum between-group difference was 1.98 pg/ml. This difference was slightly above the functional assay sensitivity (approximately 1.5 pg/ml) specified by the manufacturer.

Unlike Schütz et al. and Karanikas et al., we did not observe an elevation of serum hCT concentrations in patients with Hashimoto’s thyroiditis [18,19]. The mean and median concentrations of patients with Hashimoto’s thyroiditis were not significantly different from those obtained for the control group. Compared with the other groups, patients with Hashimoto’s thyroiditis had even the lowest mean and median concentrations. The findings presented here show that the serum hCT concentrations in our patient population were uninfluenced by the presence or absence of Hashimoto’s thyroiditis. As a result, elevated serum hCT levels were not caused by Hashimoto’s thyroiditis and generally require special diagnostic attention.

Likewise, neither the mean nor the median serum hCT concentrations of patients with nodular goitre were significantly different from those of the control group, regardless of whether the patients received or did not receive PPI therapy. For this reason, elevated serum hCT levels were not attributable to nodular goitre and require further diagnostic evaluation.

The literature reports mild or moderate increases in serum hCT concentrations after short periods of treatment with PPIs [16,17]. There were considerable differences in these increases. Vitale et al. found, for example, that gastrin responsiveness to omeprazole had great variability [17]. This can be explained by the effects of gastrin. In the present study, there were no significant differences between patients who regularly took PPIs and control subjects with a normal thyroid who did not take PPIs. The regular use of PPIs may lead to a habituation effect so that calcitonin levels are no longer elevated in response to gastrin levels. The regular use of PPIs is thus not associated with an increase in serum hCT concentrations irrespective of whether patients have a normal thyroid or present with nodular goitre. It is interesting to note, however, that patients with a healthy thyroid and PPI therapy showed an increased number of outliers. Nevertheless, an elevation of serum hCT concentrations is not necessarily attributable to the regular use of PPIs.

The present study emphasises the low susceptibility to errors of calcitonin screening. Contrary to some authors the study suggests that an elevation of hCT levels cannot be explained by conditions such as Hashimoto’s thyroiditis, nodular goitre or PPI therapy [15,16,18].

When patients have hCT levels higher than the gender-specific upper limits, the underlying cause must be thoroughly investigated. Technical problems must be ruled out. Where appropriate, a different assay should be used to perform a second measurement in order to confirm the presence of hypercalcitoninaemia [15]. An intravenous calcium stimulating test or a pentagastrin stimulation test should be performed in thoses cases [30]. A marked increase in serum hCT levels to ten times the normal level after pentagastrin or calcium stimulation is clear evidence for MTC and is an indication for thyroidectomy [3,15,30]. This approach allows the vast majority of medullary thyroid carcinomas to be detected and treated in time. Although this implies that a notable number of patients with C-cell hyperplasia, especially male patients, are likely to undergo surgery, the poor prognosis of metastatic MTC justifies this approach.

### Limitations

The results of laboratory tests might be affected by the molecular heterogeneity of calcitonin. Therefore serum calcitonin concentration can vary because different assays use antisera that recognize different epitopes of the calcitonin molecule. At present two-site immunoassays are commonly used. These tests combine monoclonal antibodies against regions, which are unique to the mature form of the calcitonin molecule. A radioisotopic (IRMA) or luminescent (ILMA) labeling is currently regarded as the most accurate [3,43]. In this study the IRMA is used. So there might be limitations in transfering the results presented here to results gathered by other assays and labelings.

The strength of the results presented here is limited by the number of subjects. Furthermore the men to women ratio is 5:1 in this study whilst worldwide the prevalence of thyroid diseases in particular Hashimoto’s thyroiditis is higher in the female then in the male sex. Therefore further studies are required to confirm the findings.

## Conclusions

Our study helps clarify the role of mildly to moderately increased calcitonin levels. The presence of Hashimoto’s thyroiditis, nodular goitre or the regular use of PPIs did not significantly influence the measured calcitonin concentrations. As a result of these findings, every above-normal increase in serum hCT levels requires particular attention and a careful evaluation since an increased production of hCT should always be suspected of indicating medullary thyroid carcinoma.

## Competing interests

The authors declare that they have no competing interests.

## Authors’ contributions

CG, AW, RS, HW devised, planned and coordinated the study. AZ was involved in the collection and analysis of the data. HW, SW assisted in interpretation of the results. CG and AW drafted the manuscript. All authors read, revised and approved the final manuscript.

## Pre-publication history

The pre-publication history for this paper can be accessed here:

http://www.biomedcentral.com/1472-6890/13/27/prepub

## References

[B1] Williams ED Brown CL Doniach I Pathological and clinical findings in a series of 67 cases of medullary thyroid carcinoma of the thyroid J Clin Pathol 1996 19 103 5909693 10.1136/jcp.19.2.103 PMC473194

[B2] Vierhapper H Niederle B Bieglmayer C Kaserer K Baumgartner-Perzer S Early diagnosis and curative therapy of medullary thyroid carcinoma by routine measurement of serum calcitonin in patients with thyroid disorders Thyroid 2005 15 11 1267 1272 10.1089/thy.2005.15.1267 16356091

[B3] Costante G Meringolo D Durante C Bianchi D Nocera M Tumino S Crocetti U Attard M Maranghi M Torlontano M Filetti S Predictive value of serum calcitonin levels for preoperative diagnosis of medullary thyroid carcinoma in a cohort of 5817 consecutive patients with thyroid nodules J Clin Endocrinol Metab 2007 92 450 455 17119000 10.1210/jc.2006-1590

[B4] Elisei R Bottici V Luchetti F Di Coscio G Romei C Grasso L Miccoli P Iacconi P Basolo F Pinchera A Pacini F Impact of routine measurement of serum calcitonin on the diagnosis and outcome of medullary thyroid cancer: experience in 10,864 patients with nodular thyroid disorders J Clin Endocrinol Metab 2004 89 163 168 10.1210/jc.2003-030550 14715844

[B5] Papi G Corsello SM Cioni K Pizzini AM Corrado S Carapezzi C Fadda G Baldini A Carani C Pontecorvi A Roti E Value of routine measurement of serum calcitonin concentrations in patients with nodular thyroid disease: a multicenter study J Endocrinol Invest 2006 29 5 427 437 16794366 10.1007/BF03344126

[B6] Rink T Truong PN Schroth HJ Diener J Zimny M Grünwald F Calculation and validation of a plasma calcitonin limit for early detection of medullary thyroid carcinoma in nodular thyroid disease Thyroid 2009 19 4 327 332 10.1089/thy.2008.0102 19355822

[B7] Regalbuto C Squatrito S La Rosa GL Cercabene G Ippolito A Tita P Salamone S Vigneri R Longitudinal study on goiter prevalence and goitrogen factors in northeastern Sicily J Endocrinol Invest 1996 19 638 645 8957750 10.1007/BF03349031

[B8] Özgen AG Hamulu F Bayraktar F Yilmaz C Tuzun M Yetkin E Tuncyurek M Kabalak T Evaluation of routine basal serum calcitonin measurement for early diagnosis of medullary thyroid carcinoma in seven hundred seventy-three patients with nodular goiter Thyroid 1999 9 6 579 582 10.1089/thy.1999.9.579 10411120

[B9] Rink T Fitz H Schroth H-J Braun S Development of the parafollicular cells in recurrent goiter Eur J Endocrinol 2001 144 485 489 10.1530/eje.0.1440485 11331214

[B10] Reid IR Cullen S Schooler BA Livingston NE Evans MC Calcitropic hormone levels in polynesians: evidence against their role in interracial differences in bone mass J Clin Endocrinol Metab 1990 70 5 1452 1456 10.1210/jcem-70-5-1452 2335580

[B11] Dibba B Prentice A Laskey MA Stirling DM Cole TJ An investigation of ethnic differences in bone mineral, hip axis length, calcium metabolism and bone turnover between West African and Caucasian adults living in the United Kingdom Ann Hum Biol 1999 26 3 229 242 10.1080/030144699282732 10355494

[B12] Saller B Görges R Reinhardt W Haupt K Janssen O Mann K Sensitive calcitonin measurement by two-site immunometric assays: implications for calcitonin-screening in nodular thyroid disease Clin Lab 2002 48 3–4 191 200 11934221

[B13] Hahm JR Lee MS Min YK Lee MK Kim KW Nam SJ Yang JH Chung JH Routine measurement of serum calcitonin is useful for early detection of medullary thyroid carcinoma in patients with nodular thyroid diseases Thyroid 2001 11 1 73 80 10.1089/10507250150500694 11272100

[B14] Pacini F Fontanelli M Fugazzola L Elisei R Romei C Di Coscio G Miccoli P Pinchera A Routine measurement of serum calcitonin in nodular thyroid diseases allows the preoperative diagnosis of unsuspected sporadic medullary thyroid carcinoma J Clin Endocrinol Metabol 1994 78 826 829 10.1210/jc.78.4.826 8157706

[B15] Dietlein M Wieler H Schmidt M Schwab R Goretzki PE Schicha H Routine measurement of serum calcitonin in patients with nodular thyroid disorders? Nuklearmedizin 2007 47 2 65 72 10.3413/nukmed-0125 18392315

[B16] Erdogan MF Gullu S Baskal N Uysal AR Kamel N Erdogan G Omeprazole: calcitonin stimulation test for the diagnosis, follow-up and family screening in medullary thyroid carcinoma J Clin Endocrinol Metab 1997 82 897 899 10.1210/jc.82.3.897 9062503

[B17] Vitale G Ciccarelli A Caraglia M Galderisi M Rossi R Del Prete S Abbruzzese A Lupoli G Comparison of Two provocative tests for calcitonin in medullary thyroid carcinoma: omeprazole vs pentagastrin Clin Chem 2002 48 9 1505 1510 12194927

[B18] Schütz M Beheshti M Oezer S Novotny C Paul M Hofmann A Bieglmayer C Niederle B Kletter K Dudczak R Karanikas G Pirich C Calcitonin measurements for early detection of medullary thyroid carcinoma or its premalignant conditions in Hashimoto’s thyroiditis Anticancer Res 2006 26 723 727 16739344

[B19] Karanikas G Moameni A Poetzi C Frequency and relevance of elevated calcitonin levels in patients with neoplastic and nonneoplastic thyroid disease and in healthy subjects J Clin Endocrinol Metab 2004 89 515 519 10.1210/jc.2003-030709 14764755

[B20] Schneider C Kobe M Schmidt M Kahraman D Malchau G Faust M Schicha H Dietlein M Calcitonin screening in patients with thyroid nodules. Diagnostic value Nuklearmedizin 2012 51 6 228 33 10.3413/Nukmed-0494-12-04 22940904

[B21] Hayashi N Tamaki N Konishi J Sonography of hashimotoβs thyroiditis J Clin Ultrasound 1986 14 123 126 45 10.1002/jcu.1870140208 3081583

[B22] Marcocci C Vitti P Cetani F Catalano F Concetti R Pinchera A Thyroid ultrasonography helps to identify patients with diffuse lymphocytic thyroiditis who are prone to develop hypothyroidism J Clin Endocrinol Metab 1991 72 1 209 13 10.1210/jcem-72-1-209 1986019

[B23] Tambouret R Szyfelbein WM Pitman MB Ultrasound-guided fine-needle aspiration biopsy of the thyroid Cancer 1999 87 5 299 305 10.1002/(SICI)1097-0142(19991025)87:5<299::AID-CNCR10>3.0.CO;2-M 10536356

[B24] Wieland M Die Krankheiten der Schilddrüse: Grundlagen 1999 Praxis: Klinik

[B25] Wiesner W Engel H Steinbrich W Oertli D Sonography of the thyroid Praxis 2006 95 15 575 80 10.1024/0369-8394.95.15.575 16640177

[B26] Medipan GmbH Medipan Arbeitsanleitung Calcitonin IRMA magnum 2008 Dahlewitz / Berlin: Medipan GmbH

[B27] Borchhardt KA Heinzl H Gessl A Hörl WH Kaserer K Sunder-Plassmann G Calcitonin concentrations in patients with chronic kidney disease and medullary thyroid carcinoma or C-cell hyperplasia Kidney Int 2006 70 11 2014 2020 17051143 10.1038/sj.ki.5001888

[B28] Kebebew E Ituarte PH Siperstein AE Duh QY Clark OH Medullary thyroid carcinoma: clinical characteristics, treatment, prognostic factors, and a comparison of staging systems Cancer 2000 88 5 1139 48 10.1002/(SICI)1097-0142(20000301)88:5<1139::AID-CNCR26>3.0.CO;2-Z 10699905

[B29] Mirallié E Iacobone M Sebag F Henry JF Results of surgical treatment of sporadic medullary thyroid carcinoma following routine measurement of serum calcitonin Eur J Surg Oncol 2004 30 7 790 795 10.1016/j.ejso.2004.05.016 15296996

[B30] Karges W Dralle H Raue F Mann K Reiners C Grussendorf M Hüfner M Niederle B Brabant B Calcitonin measurement to detect medullary thyroid carcinoma in nodular goiter: German evidence-based consensus recommendation Exp Clin Endocrinol Diabetes 2004 112 52 54 10.1055/s-2004-815727 14758572

[B31] Modigliani M Cohen R Compos JM Conte-Devolx B Maes B Boneu A Schlumberger M Bigorgne JC Dumontier P Leclerc L Corcuff B Guilhem I Prognostic factors for survival and for biochemical cure in medullary thyroid carcinoma: results in 899 patients Clin Endocrinol (Oxf) 1998 48 3 265 273 10.1046/j.1365-2265.1998.00392.x 9578814

[B32] Lips CJ Hoppener JW Thijsen JH Medullary thyroid carcinoma: the role of genetic testing and calcitonin measurement Ann Clin Biochem 2001 38 168 179 10.1258/0004563011900614 11392493

[B33] Raue F Routine calcitonin determination in thyroid nodules – an effective approach? Exp Clin Endocrinol Diabetes 1998 106 289 291 10.1055/s-0029-1211987 9792460

[B34] Dunn JT When is a thyroid nodule a sporadic meduallry carcinoma? J Clin Endocrinil Metab 1994 78 824 825 10.1210/jc.78.4.824 8157705

[B35] Horvit PK Gagel RF The goitrous patient with en elevated serum calcitonin – what to do? J Clin Endocrinol Metab 1997 82 335 337 10.1210/jc.82.2.335 9024212

[B36] Bennebaek FN Perrild H Hegedus L Diagnosis and treatment of the solitary thyroid nodule. Resuts of a European survey Clin Endocrinol (Oxf) 1999 50 357 363 10.1046/j.1365-2265.1999.00663.x 10435062

[B37] Castro R Gharib H Continuing controversy in the management of thyroid nodules Ann Intern Med 2005 42 926 931 15941700 10.7326/0003-4819-142-11-200506070-00011

[B38] Cooper DS Doherty GM Haugen BR Revised American thyroid association management guidelines for patients with thyroid nodules and differentiated thyroid cancer Thyroid 2009 19 11 1 48 19860577 10.1089/thy.2009.0110

[B39] Gharib H Papini E Paschke R American association of clinical endocrinologists, associazione medici endocrinologi, and european thyroid association: medical guidelines for clinical pratice for the diagnosis and management of thyroid nodules Endocr Pract 2010 16 1 430 20551008 10.4158/EP.16.3.468

[B40] Scheuba C Kaserer K Moritz A Drosten R Vierhapper H Bieglmayer C Haas OA Niederle B Sporadic hypercalcitoninemia - clinical and therapeutic consequences Endocr Relat Cancer 2009 16 1 243 253 18987170 10.1677/ERC-08-0059

[B41] Bevilacqua M Dominguez LJ Righini V Valdes V Toscano R Sangaletti O Vago T Baldi G Barrella M Bianchi-Porro G Increased gastrin and calcitonin secretion after oral calcium or peptones administration in patients with hypercalciuria: a clue to an alteration in calcium-sensing receptor activity J Clin Endocrinol Metab 2005 90 1489 1494 15613438 10.1210/jc.2004-0045

[B42] Vantyghem MC Danel T Marcelli-Tourvieille S Moriau J Leclerc L Cardot-Bauters C Docao C Carnaille B Wemeau JL D’Herbomez M Calcitonin levels do not decrease with weaning in chronic alcoholism Thyroid 2007 17 3 213 217 10.1089/thy.2006.0216 17381353

[B43] Rieue M Lame MC Richard A Lissak B Sambort B Vuong-Ngoc P Berrod JL Fpmbeur JP Prevelence of sporadic medullary thyroid carcinoma: the imporatance of routine measurement of serum calcitonin in the diagnostic evaluation of thyroid nodules Clin Endocrinol (Oxf) 1995 42 453 460 10.1111/j.1365-2265.1995.tb02662.x 7621562

